# Biomarkers involved in the pathogenesis of cerebral small-vessel disease

**DOI:** 10.3389/fneur.2022.969185

**Published:** 2022-09-01

**Authors:** Xiaolu Liu, Pei Sun, Jing Yang, Yuhua Fan

**Affiliations:** Department of Neurology, The First Affiliated Hospital, Sun Yat-sen University, Guangdong Provincial Key Laboratory of Diagnosis and Treatment of Major Neurological Diseases, National Key Clinical Department and Key Discipline of Neurology, Guangzhou, China

**Keywords:** cerebral small-vessel disease, biomarker, blood-brain barrier, white matter hyperintensities, lacunes, enlarged perivascular spaces, cerebral microbleeds

## Abstract

Cerebral small-vessel disease (CSVD) has been found to have a strong association with vascular cognitive impairment (VCI) and functional loss in elderly patients. At present, the diagnosis of CSVD mainly relies on brain neuroimaging markers, but they cannot fully reflect the overall picture of the disease. Currently, some biomarkers were found to be related to CSVD, but the underlying mechanisms remain unclear. We aimed to systematically review and summarize studies on the progress of biomarkers related to the pathogenesis of CSVD, which is mainly the relationship between these indicators and neuroimaging markers of CSVD. Concerning the pathophysiological mechanism of CSVD, the biomarkers of CSVD have been described as several categories related to sporadic and genetic factors. Monitoring of biomarkers might contribute to the early diagnosis and progression prediction of CSVD, thus providing ideas for better diagnosis and treatment of CSVD.

## Introduction

Cerebral small-vessel disease (CSVD) is a series of clinical, imaging, and pathological syndromes resulting from the injury of cerebral microvessels, such as 2–5 mm cerebral parenchyma around small vessels and vascular structures in the subarachnoid space (1). As a highly age-related disease, CSVD is not only closely related to vascular cognitive impairment (VCI), but also a common risk factor for depression, neurological impairment such as gait disorder, and stroke recurrence.

At present, the diagnosis of CSVD mainly depends on neuroimaging. The neuroimaging features of CSVD include recent small subcortical infarcts, lacunes, white matter hyperintensities (WMH), enlarged perivascular spaces (EPVS), cerebral microbleeds (CMB), and brain atrophy (2). Studies have shown that subcortical WMH and paraventricular WMH are present in 100 and 95%, respectively, of the elderly older than 80 years. Moreover, advanced imaging modalities, such as 7-T MRI, additional metrics, and amyloid PET provide new insights into the diagnosis and study of CSVD (3–5).

Despite the advances in neuroimaging and biological detection in recent years, the pathogenesis of CSVD remains unsolved. Blood–brain barrier (BBB) injury seems to be one of the recognized pathogenesis of sporadic CSVD (6–8), and with the discovery of genetic factors, CSVD is thought to be divided into common sporadic and rare familial forms, there are still amyloidal and non-amyloidal subtypes among sporadic forms. The amyloidal form includes cerebral amyloid angiopathy (CAA), which is considered a chronic degenerative disease characterized by multiple microbleeds (9), while the non-amyloidal is often associated with common vascular risk factors, such as hypertension. Concerning familial forms, cerebral autosomal-dominant arteriopathy with subcortical infarcts and leukoencephalopathy (CADASIL), for example, has been widely recognized. Therefore, studies on the pathological mechanism of CSVD mainly focus on sporadic and genetic types.

**Figure 1:**
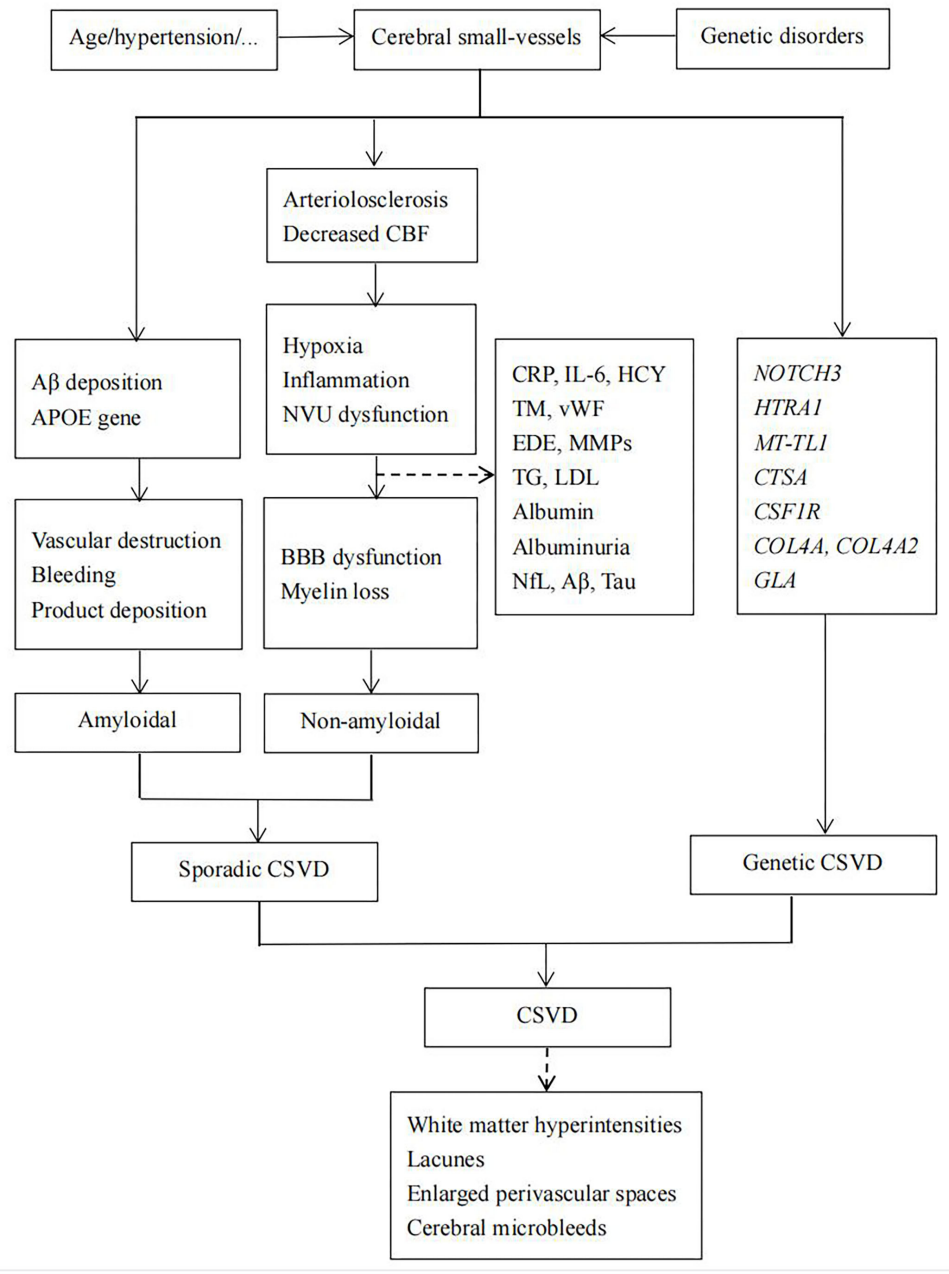
Hypothesis about the pathogenesis of cerebral small-vessel disease (CSVD). BBB, blood–brain barrier; CSVD, cerebral small-vessel disease; CBF, cerebral blood flow.

Based on the ongoing exploration of the pathogenesis of CSVD, related biomarkers have attracted attention, especially their role in the early stage of CSVD. Thus, we mainly reviewed and discussed the biomarkers involved in the pathogenesis of CSVD and their association with neuroimaging markers (Figure 1).

## Sporadic cerebral small-vessel disease

Sporadic CSVD is divided into two main forms. One of them, CAA, is a chronic degenerative disease and another one is the non-amyloid form, which is often associated with common vascular risk factors, such as old age, hypertension, diabetes, and many other vascular risk factors.

### Cerebral amyloid angiopathy

The main pathological changes of CAA are vascular destruction, bleeding, and product deposition caused by the deposition of β-amyloid protein on small arteries. Moreover, amyloid infiltration of cerebrovascular may also lead to luminal stenosis, hyaline of arterioles, intimal hyperplasia of stenosis, fibrinoid degeneration, and fibrous obstruction, resulting in focal cerebral ischemia, infarction, and softening.

Apolipoprotein E (APOE) genotype, especially APOE-ε4, a genetic marker for sporadic CAA, has been used as a genetic risk factor for CAA and Alzheimer disease (AD). APOE-ε4 was found to be associated with a high burden of EPVS in the centrum semiovale (10). While the APOE-ε2 allele appears to be more prevalent in patients with CAA-associated intracerebral hemorrhage (11).

### Non-amyloidal cerebral small-vessel disease

Non-amyloidal CSVD is less specific and usually refers to hypertensive CSVD, although it may be associated with a variety of vascular risk factors, such as diabetes. The pathological changes are mainly atherosclerosis, arteriolosclerosis, and lipohyalinosis. The exact pathogenesis remains unclear. Increased BBB permeability and endothelial dysfunction are important pathological features of sporadic CSVD. Therefore, related circulatory biomarkers may play a crucial role in the diagnosis and treatment of CSVD.

### Biomarkers of BBB and endothelial dysfunction

The BBB, which consists of endothelium, pericytes, basement membrane, and astrocytes, plays a complex and crucial role in maintaining material transport and fluid balance. In this process, at the cellular level, the endothelium is crucially important. In addition, damaged endothelial cells have been found to inhibit oligodendrocyte precursor cell maturation, which then affects the production of oligodendrocytes, leading to myelination impairment (12, 13). Dysfunction of endothelial and BBB function is usually due to chronic ischemia, inflammation, oxidative stress-induced lipid peroxidation, matrix metalloproteinase (MMP) activation, and DNA damage, and is reflected in an increase in related metabolites in blood or cerebrospinal fluid (CSF) (14). There has been a lot of evidence that these biomarkers are associated with CSVD neuroimaging markers.

#### White matter hyperintensities

##### Inflammatory biomarkers

C-reactive protein (CRP) has previously been associated with neurodegenerative diseases and poor cognitive outcomes in normal aging (15). In recent years, CRP has been found to be related to WMH severity and brain atrophy, and higher CRP levels were significantly associated with greater cognitive impairment (16–19). And in a study of 130 patients with CSVD, IL-1α and IL-6 was found a significant association with recurrent stroke and other vascular events, and there was a correlation between IL-6 and deep WMH (19, 20). Moreover, as an early marker of inflammation, procalcitonin (PCT) has previously been considered a prognostic biomarker for cardiovascular diseases (21). In recent years, Li et al. found that higher levels of PCT were closely associated with WMH (22), suggesting a monitoring role of PCT for CSVD. For vascular inflammation/endothelial dysfunction, homocysteine (HCY) has been proposed to be a risk factor, most widely investigated in conjunction with imaging burden, including WMH in patients with CSVD (23–26). Also, studies on VCAM-1 and von Willebrand factor (vWF) revealed their prominent associations with WMH (27–30).

##### Coagulation markers

It is well-known that prothrombotic status is associated with vascular risk events, and the role of coagulation biomarkers in CSVD has been increasingly discovered. A strong correlation between higher levels of thrombomodulin (TM) and WMH was found especially in the patients with microalbuminuria (31). Similarly, it was reported that higher thrombin–antithrombin values and D-dimer were associated with the presence of WMH in the previous studies (20). Moreover, vWF, synthesized by endothelial cells, was found to be related to periventricular WMH and WMH burden (32).

##### BBB integrity-related metabolites

Endothelial-derived exosomes (EDE) play a significant role in maintaining endothelial function and inflammatory regulation (33). It has been reported that plasma levels of EDE cargo proteins GLUT1, LAT1, P-GP, and NOSTRIN were significantly higher in patients with WMH, especially AD patients with WMH (34). EDE may be suggested as a biomarker associated with cerebral endothelial pathogenesis, which contributes to BBB dysfunction and degenerative changes, such as CSVD. Matrix metalloproteinases (MMPs) are involved in sustaining neuronal remodeling, BBB integrity, and have been found to be related to higher WMH grades and vascular dementia (35, 36). Among MMPs, detectable plasma matrix metalloproteinase-9 (MMP-9) is associated with the severity of CSVD and WMH (37, 38). In addition, high-density lipoprotein and triglyceride (TG) levels were found to be risk factors for new lacunes in a 3-year follow-up study (39). There were also studies that suggested that increasing triglycerides levels were associated with larger WMH volume, and increasing low-density lipoprotein (LDL) cholesterol tended to be associated with a decreased frequency and severity of MRI markers of CSVD (40). Moreover, lipoprotein-associated phospholipase A2 (LP-PLA2) was reported to be an independent risk factor associated with WMH and cognitive impairment in CSVD (41).

##### CSF and serum albumin

Elevated CSF and serum albumin that reflect albumin extravasation and BBB leakage have been found in patients with vascular dementia (42). Increased albumin CSF/serum ratio, a marker of BBB breakdown, has also been reported in patients with vascular dementia and WMH on neuroimaging (43).

#### Lacunes

A higher level of HCY has been proposed to be a risk factor most widely investigated in conjunction with the imaging burden of progression of lacunes in patients with CSVD (23–26). Li et al. also found that higher levels of PCT were closely associated with silent lacunar infarctions (22). However, no relationship was found between lacunes and CRP (24, 44). TM and fibrinogen were significantly associated with the risk of lacunes (31, 45). In addition, as the natural inhibitor of the exogenous coagulation pathway and the marker of endothelial activation, elevated factor pathway inhibitor (TFPI) were found in lacunar stroke patients than in controls, supporting the hypothesis that endothelial dysfunction is involved in the pathogenesis of lacunar stroke (46). Finally, high-density lipoprotein and triglyceride (TG) levels were found to be risk factors for new lacunes in a 3-year follow-up study (39).

#### Enlarged perivascular spaces

High levels of CRP and PCT have been reported to be associated with EPVS (22, 47, 48), and plasma HCY level was correlated with EPVS in basal ganglia (49). There is also a significant association between neutrophil count and EPVS in basal ganglia in a community-based study (50), neutrophil-to-lymphocyte ratio (NLR), and EPVS in a patient study (51). Furthermore, serum cortisol levels were found to be independent predictors of moderate-to-severe EPVS and cognitive dysfunction (52).

#### Cerebral microbleeds

Elevated levels of CRP and interleukin-6 were associated with an increased CMB burden in the stroke cohorts and more pronounced in APOE-ε4 carriers (53). Besides, higher HCY levels may result in the accumulation of amyloid protein because HCY impedes the clearance of amyloid protein through the glymphatic pathway (54), which may be the cause of its association with lobar CMB. Evidence of the studies on vascular endothelial growth factor (VEGF) also provides support for vascular inflammatory involvement in CMB formation in AD and stroke patients (55, 56).

## Genetic cerebral small-vessel disease

The discovery of genetic factors revealed a considerable impact of them on CSVD although CSVD is more often a sporadic disease. The estimated heritability for WMH as a biomarker of CSVD ranged between 50 and 80% (57). There are several hereditary forms of CSVD that have been identified, including CADASIL, cerebral autosomal recessive arteriopathy with subcortical infarcts and leukoencephalopathy (CARASIL), cathepsin A-related arteriopathy with strokes and leukoencephalopathy (CARASAL), hereditary diffuse leukoencephalopathy with spheroids (HDLS), COL4A1/2-related disorders, and Fabry disease. The numbers and in-depth investigations on the genetic loci are growing in recent years to achieve the improved diagnosis and treatment of these rare single-gene disorders, as well as sporadic CSVD (58).

Cerebral autosomal-dominant arteriopathy with subcortical infarcts and leukoencephalopathy is the most common hereditary CSVD, which is caused by a mutation of cysteine-altering in the NOTCH3 gene on chromosome 19, mainly due to mutations in the NOTCH3 extracellular domain (NOTCH3 ^ECD^). The NOTCH3 gene encodes the key molecular of transmembrane receptor protein during notch signaling and embryonal development (59), and NOTCH3 plays an essential role in the development of the vascular system in adults. There were also several reports that have described non-cysteine-related mutations and a single-particle *in vitro* aggregation assay might evaluate the clinical significance of the non-cysteine variants, but it was still a debatable point (60).

The next known rare hereditary CSVD is CARASIL, which pathogenic gene is HTRA1, located on chromosome 10q (10q25.3-q26.2). The mutation could result in the loss of HTRA1 protease activity, leading to the upregulation of TGF-β family signaling (61), resulting in the degeneration of smooth muscle cells in the cerebral small vessels.

There are several other rare hereditary forms of CSVD as follows: colony-stimulating factor 1 receptor [CSF1R, MIM^*^164770] mutations cause HDLS (62); CTSA mutations are the cause of CARASAL through the degradation of endothelin-1 and downregulation of oligodendrocyte (13); and Fabry disease is caused by the genetic mutations in the alpha-galactosidase-A gene (GLA-gene), located on the long arm of the X-chromosome (Xq22.1) (63). There is still COL4A1/2-related CSVD.

Furthermore, genome-wide association studies (GWAS) found several possible genetic factors that might be related to WMH, such as NEURL1, PDCD11, and SH3PXD2A. Mutations in TREX1, FOXC1, and PITX2 were also related to dysfunction of the vascular system, and usually present with WMH on MRI, lacunar infractions, and EPVS (64). Hence, the genetic factors play a vital role in terms of revealing the molecular mechanism of CSVD and may also bring new ideas for the prevention and treatment of CSVD.

Besides, as mentioned above, APOE-ε4 allele is associated with increased amyloid β-protein (Aβ) deposition and may lead to the formation and progression of WMH, especially in the frontal lobe. There is not only an increased risk of developing AD but also the prevalence of CAA in APOE-ε4 carries (65). Besides, Luo et al. found significantly more frontal WMH burden and basal ganglia EPVS at baseline and greater cognitive progression in APOE-ε4 carriers (66).

## Cerebral small-vessel disease associated diseases

Some of the biomarkers of CSVD are also risk factors for other diseases, including cerebral and non-cerebral diseases. Cerebral diseases are mainly neurodegenerative diseases, and non-cerebral diseases mainly include chronic kidney disease and retinal disease. They are directly or indirectly related to the pathogenesis of CSVD.

### Relationship between cerebral small-vessel disease and neurodegenerative diseases

#### White matter hyperintensities

As the significant component of the neuronal cytoskeleton, neurofilament (NfL) provides structural support for axons (67). Thus, higher serum and CSF NfL levels could be a more direct biomarker to reflect neuronal damage and neurodegeneration diseases (68), such as AD (69). CSF NfL light polypeptide was thought to be involved in increased WMH volume in dementia-free, AD, subcortical ischemic disease, and mild cognitive impairment (MCI) patients (70, 71). As the extraction of CSF is invasive, plasma NfL is more often used to evaluate the neuroaxonal damage, and serum NfL is associated with baseline WMH volume in patients with CSVD (72, 73).

CSF and plasma Aβ levels are usually used as the biomarkers of AD, and the toxic effects of Aβ on blood vessel walls are also causing concern. There have been several studies investigated the association between Aβ and WMH (74) in which Gurol et al. reported an association between WMH and plasma Aβ40 and Aβ42 (75), while Kester et al. found a negative correlation between CSF Aβ42 and WMH in a normal population (76), indicating Aβ a potential risk factor for CSVD. Similarly, some studies also found the relationship between WMH and CSF total tau (t-tau) and phosphorylated tau (p-tau) (76–78).

#### Lacunes

Serum NfL is found to be associated with the baseline presence of lacunes in CSVD patients (72, 73). In CSVD patients with higher levels of Aβ1-42, CRP was strongly associated with lacunes (47).

#### Enlarged perivascular spaces

Higher levels of P-Tau, T-Tau, and neurogranin in Aβ-positive individuals were significantly associated with EPVS in centrum semiovale (79). And CRP was strongly associated with EPVS in CSVD patients with higher Aβ1-42 (47).

#### Cerebral microbleeds

The previous studies have found that the CMB is related to low CSF Aβ42, but there were also studies found that there was no correlation between CSF Aβ42 and deep CMB (80, 81). It's worth noting that Kester et al. found that CMB was related to CSF Aβ42 only in the presence of APOE ε4 in AD and normal elderly (76). And there is a significant association between CMB and tau pathology that is lower tau and p-tau 181 in APOE ε4 non-carriers, but not in carriers (76, 82, 83).

### Relationship between cerebral small-vessel disease and non-cerebral diseases

Some extracranial diseases have also been found to have vascular lesions similar to those in CSVD, such as arteriosclerosis and endothelial dysfunction. The most common is chronic kidney disease (CKD). It has been reported that patients with CKD are more likely to develop CSVD and more severe WMH, but the pathological mechanism of CKD-related CSVD remains unclear and might be related to uremic toxins and chronic inflammation (84–86). As the main feature of CKD, albuminuria has been proposed as an independent biomarker for systemic endothelial dysfunction (87). There is also evidence suggesting an association between albuminuria and WMH burden (88), and another indicator of kidney function, lower GFR, could also reflect the advanced stage of microvascular disease (89). In addition, hyperphosphatemia was found to be significantly associated with vascular risk events (90, 91). In recent years, Chung et al. reported that higher circulatory phosphate levels were associated with severe WMH and downregulating tight junction proteins in human brain microvascular endothelial cells (92), suggesting that hyperphosphatemia might be a novel risk factor for CSVD and might be involved in BBB impairment. As the early marker of kidney disease, albuminuria has been proposed as an independent biomarker for systemic endothelial dysfunction (87). It's pretty clear that worse kidney function, associated with peripheral systemic microvascular disease, has been the biomarker that could be useful in the evaluation of brain microvascular damage.

On the other hand, the vascular network of the retina is physiologically similar to the corresponding cerebral neurovascular units (93). Therefore, non-invasive evaluation of retinal neurons and blood vessels may provide new biomarkers for the diagnosis and evaluation of CSVD (94, 95). Optical coherence tomography (OCT) showed a significant correlation between the Wall to Lumen Ratio (WLR) and WMH (96), and OCT angiography (OCTA) showed that retinal hypoperfusion was related to MRI markers, such as WMH and lacunes in CSVD patients (97), suggesting the potential value that these parameters as biomarkers for early CSVD.

## Other biomarkers

Renin–angiotensin–aldosterone System: The renin–angiotensin–aldosterone system previously has been studied as a potential marker in CSVD because it works in the regulation of vascular smooth muscle constriction and hypertension (98). And angiotensin-II, the key molecular related to hypertension, is found to be involved in BBB damage in CSVD (99–101). Specifically, increased angiotensin-converting enzyme (ACE) levels have been found in the patients with greater progression of deep WMH volume but less progression of cortical atrophy, suggesting a complex role of ACE in the brain (102).

Plasma Brain Natriuretic Peptide (BNP) and NT-proBNP: BNP and NT-proBNP are considered the diagnostic markers of cardiovascular diseases and have been linked to cerebrovascular diseases in recent years (103). Increased levels of plasma BNP are associated with WMHs and lacunar infarcts, but there was a negative correlation between BNP and CMB (104), thus it could be a useful biomarker for identifying ischemic CSVD in patients with hypertension. In addition, Vilar-Bergua et al. also found a higher level of NT-proBNP was independently associated with silent brain infarcts, CMB, EPVS, and WMHs volumes (103). The possible mechanism is that BNP reduces local blood flow and blood pressure, thereby reducing cerebral blood flow and causing ischemic injury.

## Conclusion

Despite the severe disease burden of CSVD, its pathologic mechanisms are not fully understood by clinicians. The basis for diagnosis and treatment of CSVD is mainly derived from neuroimaging, such as diffusion tensor imaging, imaging of the BBB, cerebrovascular reactivity, and cerebral blood flow, which partly reflect the pathological mechanisms of sporadic CSVD, such as BBB damage, reduced blood flow, and increased intracranial vascular pulsation (4, 105). And the advancement of molecular genetic tests improves diagnostic accuracy in patients with potential CSVD. Therefore, most studies focus on the sporadic and genetic types of CSVD.

Study on biomarkers of CSVD has become a promising field in disease diagnosis and monitoring. Numerous studies have suggested that APOE genotype is associated with amyloid angiopathy in sporadic CSVD, and biomarkers suggesting BBB damage, such as inflammatory factors and coagulation factors, are closely related to non-amyloidosis subtypes, especially their close association with neuroimaging markers. In addition, molecular testing helps us to improve the detection rate of hereditary CSVD. Moreover, there are also some diseases with pathological changes similar to CSVD, such as neurodegenerative diseases and renal diseases. These known biomarkers reflect the involvement of several interrelated pathways including but not limited to endothelial and BBB dysfunction and genetic factors, showing the strong association between the possible biomarkers and CSVD. But their predictive or discriminative ability regarding diagnosis remains to be established perfectly during clinical research.

In summary, although the diagnosis of CSVD is still mainly relied on neuroimaging, the study of biomarkers, especially their association with neuroimaging markers, can help us better early identification, prediction, and evaluation of the development of CSVD, and may find new therapeutic targets.

## Author contributions

XL and YF designed the study. XL, PS, and JY collected the data. XL drafted the manuscript. All authors approved the final version of the manuscript.

## Funding

This study was supported by grants from the National Natural Science Foundation of China (No. 82071294), Guangdong Provincial Key Laboratory of Diagnosis and Treatment of Major Neurological Diseases (2020B1212060017), Guangdong Provincial Clinical Research Center for Neurological Diseases (2020B1111170002), the Southern China International Cooperation Base for Early Intervention and Functional Rehabilitation of Neurological Diseases (2015B050501003 and 2020A0505020004), and Guangdong Provincial Engineering Center for Major Neurological Disease Treatment, Guangdong Provincial Translational Medicine Innovation Platform for Diagnosis and Treatment of Major Neurological Disease.

## Conflict of interest

The authors declare that the research was conducted in the absence of any commercial or financial relationships that could be construed as a potential conflict of interest.

## Publisher's note

All claims expressed in this article are solely those of the authors and do not necessarily represent those of their affiliated organizations, or those of the publisher, the editors and the reviewers. Any product that may be evaluated in this article, or claim that may be made by its manufacturer, is not guaranteed or endorsed by the publisher.
